# Unconventional Immunotherapies in Cancer: Opportunities and Challenges

**DOI:** 10.3390/ph18081154

**Published:** 2025-08-04

**Authors:** Meshael Alturki, Abdullah A. Alshehri, Ahmad M. Aldossary, Mohannad M. Fallatah, Fahad A. Almughem, Nojoud Al Fayez, Majed A. Majrashi, Ibrahim A. Alradwan, Mohammad Alkhrayef, Mohammad N. Alomary, Essam A. Tawfik

**Affiliations:** 1 Wellness and Preventative Medicine Institute, Health Sector, King Abdulaziz City for Science and Technology (KACST), Riyadh 11442, Saudi Arabia; mmsalturki@kacst.gov.sa (M.A.); mfallatah@kacst.gov.sa (M.M.F.); falmughem@kacst.gov.sa (F.A.A.); nalfayez@kacst.gov.sa (N.A.F.); 2 Advanced Diagnostics and Therapeutics Institute, Health Sector, King Abdulaziz City for Science and Technology (KACST), Riyadh 11442, Saudi Arabia; abdualshehri@kacst.gov.sa (A.A.A.); aaldossary@kacst.gov.sa (A.M.A.); ialradwan@kacst.gov.sa (I.A.A.); malomary@kacst.gov.sa (M.N.A.); 3 Bioengineering Institute, Health Sector, King Abdulaziz City for Science and Technology (KACST), Riyadh 11442, Saudi Arabia; mmajrashi@kacst.gov.sa; 4 Disability Research Institute, Health Sector, King Abdulaziz City for Science and Technology (KACST), Riyadh 11442, Saudi Arabia; mkhuryef@kacst.gov.sa

**Keywords:** unconventional immunotherapy, γδ T cells, iNKT cells, MAIT cells, bispecific antibodies, immune engineering, synthetic biology

## Abstract

Conventional immunotherapy, including immune checkpoint blockade and chimeric antigen receptor (CAR)-T cells, has revolutionized cancer therapy over the past decade. Yet, the efficacy of these therapies is limited by tumor resistance, antigen escape mechanisms, poor persistence, and T-cell exhaustion, particularly in the treatment of solid tumors. The emergence of unconventional immunotherapies offers novel opportunities by leveraging diverse immune cell subsets and synthetic biologics. This review explores various immunotherapy platforms, including gamma delta T cells, invariant natural killer T cells, mucosal-associated invariant T cells, engineered regulatory T cells, and universal CAR platforms. Additionally, it expands on biologics, including bispecific and multispecific antibodies, cytokine fusions, agonists, and oncolytic viruses, showcasing their potential for modular engineering and off-the-shelf applicability. Distinct features of unconventional platforms include independence from the major histocompatibility complex (MHC), tissue-homing capabilities, stress ligand sensing, and the ability to bridge adaptive and innate immunity. Their compatibility with engineering approaches highlights their potential as scalable, efficient, and cost-effective therapies. To overcome translational challenges such as functional heterogeneity, immune exhaustion, tumor microenvironment-mediated suppression, and limited persistence, novel strategies will be discussed, including metabolic and epigenetic reprogramming, immune cloaking, gene editing, and the utilization of artificial intelligence for patient stratification. Ultimately, unconventional immunotherapies extend the therapeutic horizon of cancer immunotherapy by breaking barriers in solid tumor treatment and increasing accessibility. Continued investments in research for mechanistic insights and scalable manufacturing are key to unlocking their full clinical potential.

## 1. Introduction

Immunotherapy has reshaped cancer therapy, driven by the clinical success of immune checkpoint inhibitors and chimeric antigen receptor (CAR)-T cell therapies [1,2]. The first approved checkpoint inhibitor was cytotoxic T-lymphocyte-associated protein 4 (CTLA-4), which showed successful clinical responses in multiple cancers [3], followed by programmed cell death protein 1 (PD-1) blockade that had long-lasting responses clinically [4]. Combining both CTLA-4 and PD-1 showed even greater success clinically [1]. Similarly, CAR-T cells have shown curative promise in blood cancers [5]. However, both have significant restrictions, particularly with solid tumors [6].

Only a small proportion of patients benefit from immune checkpoint inhibitors (ICIs) due to factors such as tumor immune escape, lack of neoantigens, and immunotoxicity [7]. Another key limitation is the absence of tumor-infiltrating lymphocytes (TILs) in many tumors. These “cold” tumors lack the pre-existing immune activity required for ICIs to be effective, as there are few T cells to reactivate [8]. In contrast, “hot” tumors, which are rich in TILs, tend to respond better. Strategies to convert cold tumors into hot ones are being explored to enhance ICI efficacy [9,10]. The successful use of CAR-T therapies is somewhat limited by antigen heterogeneity, T-cell dysfunction and exhaustion, manufacturing challenges, and toxicities, including cytokine release syndrome [11].

To overcome these limitations, the field has begun to explore non-conventional immunotherapies, an expanding class of therapeutics that use non-canonical immune cells, such as gamma delta (γδ) T cells, invariant natural killer T cells (iNKT), mucosal-associated invariant T cells (MAIT), and engineered regulatory T cells (Tregs), combined with novel biologics, including bispecific T-cell engagers (BiTEs), multi-specific antibodies, and oncolytic viruses [12]. Such strategies provide major histocompatibility complex (MHC)-unrestricted recognition, tissue residency, and fast effector responses, effectively adding solid tumors and immune-related cancers to the ranks of immune therapy targets [13].

As the field of immuno-oncology continues to mature, these non-standard therapies are expected to reshape therapeutic landscapes by providing off-the-shelf, universally applicable, and synthetically designed solutions to circumvent current roadblocks. This review summarizes the main platforms, immunological foundation, translational hurdles, and perspectives of unconventional immunotherapies, emphasizing opportunities and challenges that will define the future of immunotherapies.

## 2. Unconventional Cell-Based Therapies

Unconventional T cells represent a unique and functionally diverse subset of lymphocytes distinguished by their specialized T-cell receptors (TCRs) and ability to recognize antigens through non-classical, MHC-independent pathways. Unlike conventional alpha-beta (αβ) T cells that depend on peptide antigens presented by polymorphic MHC molecules, unconventional T cells, including iNKT cells, γδ T cells, and MAIT cells, are activated by a distinct set of ligands. These include metabolite-derived antigens presented by CD1d, a member of the CD1 glycoprotein molecule family, for recognition by iNKT cells, phosphoantigens sensed through the butyrophilin 3A1/2A1 complex by γδ T cells (particularly Vγ9Vδ2, the gamma 9 delta 2 subtype of gamma delta T-cells), and microbial riboflavin metabolites presented by MHC class I-related protein 1 (MR1) for recognition by MAIT cells. Their broad tissue distribution, particularly at barrier sites and in mucosal tissues, allows them to mount rapid and localized immune responses against malignant or stressed cells. Functionally, these cells bridge innate and adaptive immunity, exerting cytotoxic activity and producing cytokines such as interferon gamma (IFN-γ) and tumor necrosis factor-alpha (TNF-α) through both TCR-mediated and natural killer (NK) receptor-mediated signaling pathways. Importantly, they have demonstrated the capacity to infiltrate solid tumors and modulate the immunosuppressive tumor microenvironments, and because of their MHC-independent antigen recognition and low alloreactivity, they are associated with reduced risk of graft-versus-host disease (GvHD), making them especially attractive for allogeneic cell therapies and engineering platforms such as CAR constructs [14,15,16,17,18]. Universal CAR strategies aim to overcome limitations of autologous therapies by leveraging unconventional cells or gene editing to minimize immunogenicity. Platforms based on γδ T cells, iNKT cells, and NK cells, often modified to lack human leukocyte antigen (HLA) expression, reduce the risk of rejection and GvHD. Clinical trials using hypoimmunogenic CAR-T cells (e.g., HLA-I/II-deficient alloCARs) have shown favorable safety profiles and early signs of efficacy. These platforms enable scalable, immediate access to therapy, addressing urgent clinical needs in relapsed hematologic malignancies and solid tumors [18].

### 2.1. Invariant Natural Killer T Cells

iNKT cells possess innate-like cytotoxic functions and a unique ability to infiltrate solid tumors. CAR-iNKT cells targeting disialoganglioside (GD2) [19], B-lymphocyte antigen (CD19) [20], or B-cell maturation antigen (BCMA) [21] have demonstrated potent activity across models of neuroblastoma, B-cell lymphomas, and multiple myeloma, respectively. A Phase I trial using autologous GD2-targeted, Interleukin (IL)-15 (i.e., IL-15)-secreting CAR-iNKT cells in pediatric neuroblastoma demonstrated a 25% objective response rate, including one complete remission, with no dose-limiting toxicities [19]. Future strategies are required to enhance the efficacy of iNKT-based therapies, such as donor selection for CD62L^+^ (cell adhesion molecule) CAR iNKT cell subsets, which are known for their robustness in in vivo expansion, and the use of IL-12 to promote type 1 helper (Th1)-polarized, exhaustion-resistant phenotypes [22]. Importantly, their TCR recognition of non-polymorphic CD1d limits alloreactivity, positioning iNKT cells as promising off-the-shelf therapeutic platforms [14]. 

### 2.2. Mucosal-Associated Invariant T Cells

MAIT cells reside primarily in mucosal tissues and blood, recognizing microbial vitamin B metabolites via MHC class I-like molecule MR1. They exhibit robust cytotoxicity and NK receptor expression (e.g., NKG2D, DNAM-1 (DNAX accessory molecule 1)) and possess an effector-memory T-cell phenotype [15]. MAIT cells have emerged as positive prognostic indicators in hepatocellular carcinoma (HCC), non-small-cell lung cancer (NSCLC), and ovarian cancer, particularly in response to immune checkpoint inhibitors and platinum-based chemotherapy [23,24,25]. In HCC, they synergize with anti-PD-L1 therapy by targeting tumor-associated macrophages through PD-1/PD-L1 interactions. In NSCLC, CXCR6+ (CXC motif chemokine receptor 6) MAIT cells have also shown synergy with anti-PD-1 therapy. Early preclinical studies using CAR-MAIT cells show encouraging in vitro efficacy [26]. However, challenges remain in using MAIT cells, particularly in their ex vivo expansion and engineering.

### 2.3. Gamma Delta T Cells

Gamma delta (γδ) T cells combine features of adaptive and innate immunity, playing essential roles in stress surveillance and tumor immunity. They recognize phosphoantigens and stress ligands without MHC restriction. The Vδ2^+^ (delta 2 subset of gamma delta T cells) subset is the most prominent in blood. It detects phosphoantigens resulting from mevalonate pathway dysregulation in tumor cells, while Vδ1^+^ (delta 1 subset of gamma delta T cells) subsets dominate mucosal sites and target non-classical MHC molecules, such as CD1 (family of glycoproteins) and MR1 (MHC class I-related protein 1) [16,27]. Elevated γδ T-cell levels correlate with favorable outcomes post-hematopoietic stem cell transplantation [28]. Clinical trials using CAR-γδ T cells have reported moderate success, with barriers including short persistence and donor variability limiting their efficacy. Engineered CD16^+^ (low-affinity Immunoglobulin G receptor) γδ T cells with CARs and IL-15 showed enhanced cytotoxicity and tumor control in ovarian cancer and glioblastoma models [29,30].

### 2.4. Double-Negative T Cells and Engineered Regulatory T Cells

Double-negative T cells (CD4^−^CD8^−^, co-receptors for the T-cell receptor) are rare but exhibit both cytotoxic and regulatory potentials. Recent studies have shown that they eliminate leukemic cells via a tumor necrosis factor α-Janus kinase 1-Intercellular Adhesion Molecule 1 (TNFα–JAK1–ICAM-1) axis without inducing GvHD, highlighting their potential in adoptive immunotherapy [31]. Engineered regulatory T cells (CAR-Tregs) are advancing as targeted immunomodulators in autoimmunity and transplantation. Preclinical data indicate that CAR-Tregs can induce antigen-specific tolerance and mitigate inflammation. Current efforts focus on enhancing the in vivo stability and suppressive functionality of these agents through synthetic biology tools [17,32].

**Figure 1:**
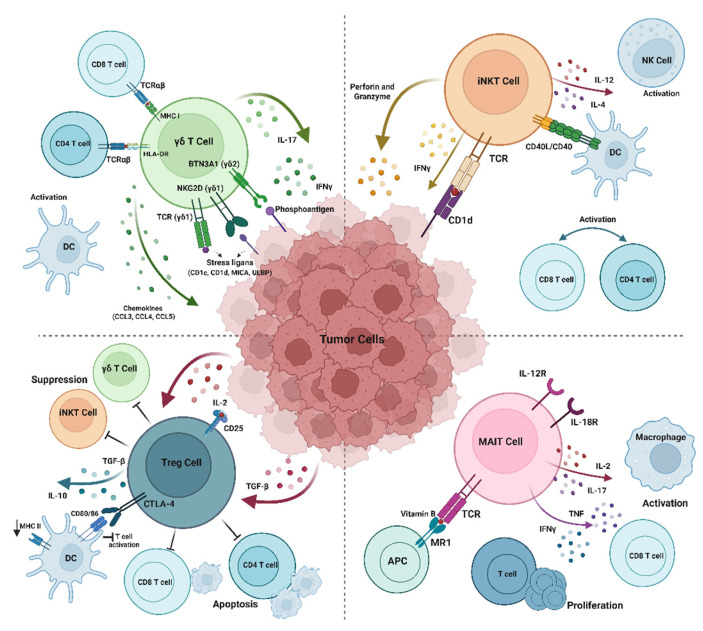
Interplay between unconventional and conventional immune cells in the tumor microenvironment. Created with BioRender.com.

Figure 1 highlights the cellular crosstalk shaping immune responses within the tumor microenvironment. Unconventional T cells, including iNKT cells, γδ T cells, and MAIT cells, respond to non-classical antigens or stress signals and secrete cytokines that activate dendritic cells, enhance antigen presentation, and support the recruitment and function of αβ T cells. MAIT cells can also be activated by inflammatory signals such as IL-12 and IL-18, further amplifying local immune activity. In contrast, regulatory T cells help maintain suppression by limiting dendritic cell maturation and limiting both αβ and unconventional T-cell activity through the expression of IL-10, transforming growth factor-β (TGF-β), and CTLA-4 as well as the consumption of IL-2 [13,33].

## 3. Unconventional Biologics

This section focuses on the unconventional biologics for cancer therapy, aiming to describe bispecific and trispecific engagers, multispecific antibodies, cytokine fusions and agonists, as well as oncolytic viruses and synthetic payload systems. Regarding bispecific and trispecific engagers, they are antibodies engineered to act as immune cell engagers. They typically possess at least one arm that binds to a tumor-associated antigen and another that targets a receptor on immune effector cells to activate them [34]. For instance, bispecific and trispecific T-cell engagers (BiTEs and TriTEs) are proteins that activate T-cell immunity independently of antigen-presenting cells [35]. A well-known example is blinatumomab, a BiTE used for the treatment of acute lymphoblastic leukemia. It consists of two linked antibody fragments—one binds to the CD19 antigen on B cells and the other to the CD3 (T-cell co-receptor) antigen on T cells. This bridging induces T cells to release granzymes and perforins, leading to the lysis of B cells [36]. While BiTEs like blinatumomab have shown therapeutic success, limitations such as treatment resistance and limited activity against solid tumors have been observed. To address these issues, trispecific antibodies were developed. Compared to BiTEs, TriTEs have an extended function, as they can simultaneously target three antigens—for example, T-cell receptors, tumor antigens, and NK cell activators [37].

Regarding multispecific antibodies, they have several advantages over BiTEs and TriTEs. They can target more than three antigens simultaneously, which enhances their effectiveness against tumor heterogeneity by engaging a broader array of tumor-associated antigens. Furthermore, their design allows them to overcome resistance seen with monospecific antibodies, as they can bind multiple target sites on the surface of cancer cells concurrently [38]. For the cytokine fusions treatment approach, cytokines are potent regulators of the immune system [39,40]. Preclinical studies have demonstrated their anticancer effects when used alone or in combination [41,42]. However, their clinical use is limited due to their high toxicity. Although IL-12 has shown strong antitumor effects in mouse models, its safe dosage in humans is ≤1 μg/kg, which restricts its application [43,44]. To reduce toxicity while maintaining efficacy, strategies such as the creation of immunocytokines have been explored. These involve fusing cytokines to tumor-targeting antibodies, thereby enhancing cytokine selectivity and enabling targeted delivery to tumor sites [45].

Oncolytic viruses and synthetic payload systems can be used for the treatment of cancers. Oncolytic viruses—either naturally occurring or genetically engineered—can selectively target and destroy cancer cells [46,47]. This is achieved via multiple mechanisms. First, they infect and replicate within cancer cells, causing cell lysis. This releases new viral particles that spread to neighboring tumor cells, amplifying the effect [48]. Additionally, the lysis of cancer cells releases tumor-associated antigens and elevates cytokine levels, stimulating the immune system and potentially enhancing the effectiveness of immune checkpoint inhibitors [49]. Moreover, oncolytic viruses can be engineered to deliver therapeutic genes directly into the tumor microenvironment [50]. For instance, Talimogene laherparepvec (T-VEC), a herpes simplex virus modified to carry the gene for granulocyte-macrophage colony-stimulating factor (GM-CSF), induces infected tumor cells to produce GM-CSF, thereby boosting local immune responses [51].

## 4. Distinct Immune Features of Unconventional Platforms

Unconventional immunotherapeutic platforms incorporate a variety of immunological effectors that go beyond standard processes that rely on TCRs or B-cell receptors (BCR) [52]. This sort of immunological response has been linked to innate-like lymphocytes, MAIT cells, γδ T cells, iNKT cells, NK cells, and other innate lymphoid cells (ILCs) [53]. These cell types all have distinct immunological characteristics that enable tumor identification and reaction without the need for traditional MHC presentation. They are, therefore, excellent prospects for cancer immunotherapies in the future [54]. This Section 5 examines these platforms’ significance for therapeutic innovation and identifies the immunological characteristics that distinguish them from traditional methods.

### 4.1. MHC-Independence

A hallmark of unconventional immune effectors is their ability to recognize and eradicate tumor cells independently of classical MHC molecules, a critical advantage in malignancies where MHC class I downregulation enables tumors to elude CD8^+^ T-cell detection [55]. For example, γδ T cells, particularly the Vγ9Vδ2 subset, bypass peptide-MHC restriction entirely by sensing small, non-peptidic phosphoantigens (e.g., isopentenyl pyrophosphate, IPP) via butyrophilin (BTN) molecules. Specifically, BTN3A1 senses intracellular accumulation of phosphoantigens and undergoes a conformational change that is detected by BTN2A1 on the cell surface, allowing stable interaction with the γδ TCR [56]. Similarly, iNKT cells recognize glycolipid antigens (e.g., α-GalCer analogs) presented by the non-classical MHC molecule CD1d. This allows iNKT cells to bridge innate and adaptive immunity by rapidly secreting cytokines and directly lysing target cells. Similarly, MAIT cells detect microbial riboflavin metabolites, allowing them to respond to infection or dysbiosis-associated tumors independently of polymorphic HLA molecules [57]. NK cells, on the other hand, use a “missing-self” strategy whereby reduced or absent MHC class I expression disrupts inhibitory signaling (mediated by receptors like Killer-cell immunoglobulin-like receptors (KIRs) or NKG2A (a checkpoint molecule), tipping the balance toward activation and target cell lysis. NK cells also express activating receptors (e.g., NKG2D, DNAM-1, NKp30 (a stimulatory receptor)) that detect stress-induced ligands commonly upregulated on malignant cells, further enhancing tumor surveillance [58]. This MHC-independent targeting lessens the vulnerability to immune evasion by tumors and broadens therapeutic applicability across diverse patient populations, as efficacy is not limited by HLA variability. Furthermore, these cells circumvent central tolerance restrictions, enabling recognition of a wider array of tumor-associated antigens compared to conventional T cells [59].

### 4.2. Stress Ligand and Metabolite Sensing

The capacity of unconventional immune platforms to identify stress-induced ligands and metabolic changes, important markers of malignant transformation, is one of their distinguishing characteristics. This makes it possible for these cells to identify and react to malignancies even when traditional antigen presentation is not present [60]. Stress-related molecules, including MHC class I chain-related protein A and B (MICA/B) and Novel MHC Class I-Related Molecules (ULBPs), which are often increased on tumor cells and interact with the activating receptor NKG2D [61], are especially receptive to NK cells and γδ T cells. A significant subgroup of γδ T cells, namely Vγ9Vδ2 T cells, is also susceptible to the build-up of isoprenoid pathway intermediates, such as isopentenyl pyrophosphate, a metabolic marker frequently seen in transformed cells [62]. Likewise, MAIT cells can respond to metabolic dysregulation and tumor-associated microbial imbalances by identifying microbial and tumor-derived compounds that are presented by MR1. These unique sensing capabilities expand the spectrum of tumor recognition, offering strategies to target immunologically “cold” tumors that evade conventional immune responses. Additionally, they facilitate early detection and interaction in tumor microenvironments that are high in stress, which may result in more efficient immune activation [63,64].

### 4.3. Reduced Risk of GvHD and Alloreactivity

Because alloantigens are recognized in the context of MHC, traditional TCR-based treatments, especially those using allogeneic T cells, carry a significant risk of GvHD [65]. On the other hand, a lot of non-traditional immune effectors have limited alloreactive potential, which makes them ideal for generic, off-the-shelf uses [66]. Because of their reduced reliance on alloantigen specificity and MHC-independent recognition mechanisms, γδ T cells and NK cells have demonstrated a minimal capacity to generate GvHD in both preclinical and clinical contexts [67]. For instance, allogeneic NK cell treatments are being demonstrated for use in solid tumors and have been safely used in hematologic malignancies [68]. The semi-invariant TCRs used by MAIT and iNKT cells, which identify antigens presented by non-polymorphic molecules (MR1 and CD1d, respectively), also exhibit intrinsically decreased alloreactivity. These characteristics support the development of universal donor cell therapies and the creation of standardized, banked immune cell products [69]. Such approaches can streamline manufacturing, reduce costs, and address logistical challenges typically associated with autologous cell therapies.

### 4.4. Immune Bridging Functions

Unconventional immune cells are essential for coordinating the interactions between innate and adaptive immunity in addition to their direct cytotoxicity. Their immunomodulatory properties make them a valuable therapeutic tool. When activated, iNKT cells release many cytokines (including IFN-γ and IL-4) that boost CD8^+^ T-cell activation, improve antigen presentation, and encourage dendritic cell maturation [70]. Likewise, γδ T cells, namely the Vδ1 fraction, have the ability to take on context-dependent characteristics, which can aid in immune priming or control in the tumor microenvironment [64]. MAIT cells can enhance antitumor responses by promoting localized inflammation through the production of IFN-γ, TNF-α, and IL-17. These immunoregulatory characteristics can be used to improve the effectiveness of immunotherapies such as checkpoint inhibitors and cancer vaccines and to reprogram immunosuppressive tumor microenvironments [15].

### 4.5. Tissue Homing and Residency

Unconventional lymphocytes exhibit unique tissue homing and residence patterns that correspond with the anatomical locations of numerous solid tumors and facilitate ongoing local immune monitoring. Because certain homing receptors (such as Chemokine receptor 6 (CCR6), CXCR6, and chemokine receptor 9 (CCR9)) are expressed, MAIT and γδ T cells are more abundant in epithelial and mucosal tissues, such as the skin, gut, liver, and lung [64]. Tissue residency is another feature of iNKT and some NK cell subsets; populations that remain in the liver show significant anticancer potential. Direct tumor infiltration is made possible by this strategic localization, which also aids in the development of targeted treatments [71].

### 4.6. Rapid Effector Function

Immediate cytotoxic and cytokine-mediated responses that avoid the lag phase typical of traditional adaptive immunity are made possible by innate-like cells, which are ready for instant activation [72]. Cytotoxic granules that are pre-formed with perforin and granzymes are stored by NK cells, enabling quick death upon engagement [73]. MAIT cells contribute to early immune activation by rapidly responding to metabolic signals, while γδ T cells and iNKT cells also exhibit fast-acting effector responses. With prompt intervention, this quick capability can potentially reverse immune evasion and is particularly beneficial in high-burden or rapidly developing tumors [74].

### 4.7. Suitability for Engineering

Unconventional immune cells are prime candidates for synthetic alteration, such as CAR engineering, bispecific targeting, and gene editing, due to their innate-like traits and restricted receptor diversity [66]. The CAR-NK and CAR-γδ T-cell systems, which combine the accuracy of antigen-specific targeting with the safety and speed of innate immunity, have shown promising preclinical and early clinical performance [66]. Additionally, engineered MAIT and iNKT cells exhibit promise for use in immune-privileged and mucosal tissues. Furthermore, sophisticated techniques like synthetic cytokine circuits, switchable adaptors, and inducible gene systems improve function and controllability, increasing persistence and lowering the possibility of off-target effects or depletion [75].

Unconventional immune platforms offer a unique and powerful alternative to classical TCR-based immunotherapies. Their MHC-independent recognition, stress and metabolite sensing, low alloreactivity, immunomodulatory capacity, tissue tropism, rapid effector function, and engineering compatibility position them as a versatile and promising class of cancer therapeutics [76]. Continued clinical and technological progress is likely to accelerate their integration into both personalized and off-the-shelf treatment paradigms across a wide range of malignancies [77].

## 5. Translational Challenges

Unconventional immunotherapies, including those that utilize γδ T cells, MAIT cells, invariant NKT cells, and engineered regulatory T cells, offer promising avenues for cancer treatment [66]. However, their clinical translation faces several significant challenges. One of the key translational challenges of using unconventional immune cells, such as γδ T cells, lies in their low abundance in peripheral blood, making their isolation technically demanding. In addition, the ex vivo expansion of these cells requires specialized and highly defined culture conditions, which often result in suboptimal fold expansion. These limitations hinder large-scale manufacturing and clinical translation, emphasizing the need for optimized protocols and alternative sources, such as cord blood or induced pluripotent stem cells (iPSCs), to overcome scalability issues.

### 5.1. Phenotypic and Functional Heterogeneity

Unconventional immune cells demonstrate substantial phenotypic and functional diversity, complicating their therapeutic application. For instance, γδ T cells can be categorized into Vδ1 and Vδ2 subsets, each with distinct tissue distributions and functional properties [64]. Vδ1 cells are predominantly tissue-resident, while Vδ2 cells circulate in the blood (75% or more of the γδ T-cell population) and respond to phosphoantigens [64,78]. This subset-specific behavior affects their expansion and persistence post transfer. For instance, Vδ2 T cells can be expanded ex vivo using aminobisphosphonates like zoledronate, but the in vivo persistence remains limited due to factors such as activation-induced cell death and lack of homeostatic cytokines [79]. 

Moreover, the functional plasticity of γδ T cells can lead to divergent outcomes; while some subsets exhibit potent antitumor activity through IFN-γ production, others may promote tumor progression via IL-17 secretion [79,80,81]. Similarly, MAIT cells show diverse cytokine profiles and activation states, depending on the tumor microenvironment (TME), which in turn influences their cytotoxic potential. They can demonstrate a range of functional states, from cytotoxic effector cells to regulatory phenotypes. This functional heterogeneity complicates their therapeutic use, as expanding cells with desired antitumor properties can be challenging. Such variability necessitates precise characterization and selection of cell subsets for therapeutic use [81,82].

### 5.2. Poorly Defined Exhaustion in Unconventional Immune Cells

While T-cell exhaustion is well-characterized in conventional αβ T cells, marked by upregulation of inhibitory receptors like PD-1, mucin domain-containing protein 3 (Tim-3), and lymphocyte-activation gene 3 (LAG-3), the exhaustion profiles of unconventional immune cells remain less understood [83,84,85]. Emerging evidence suggests that γδ T cells and MAIT cells can also express exhaustion markers under chronic stimulation, leading to diminished effector functions [85]. For instance, a tuberculosis infection can impair the Signal transducer and activator of transcription 3/Janus kinase 2 (STAT3/JAK2) signaling axis in Vδ2 T cells, a subset of γδ T cells, leading to exhaustion when stimulated with IL-23 and the phosphoantigen HMBPP ((*E*)-4-Hydroxy-3-methyl-but-2-enyl pyrophosphate) [85,86]. This exhaustion is characterized by a loss of proliferative and cytokine-producing capacity [86]. However, this impairment cannot be reversed by blocking PD-1, suggesting an alternative exhaustion mechanism that differs from that of αβ T cells [85,86]. This can delay the development of strategies to reverse exhaustion and enhance their therapeutic efficacy.

### 5.3. Suppression by the Tumor Microenvironment (TME)

The TME poses a formidable barrier to effective immunotherapy. It is characterized by hypoxia, acidic pH, immunosuppressive cytokines (e.g., TGF-β and IL-10), and the presence of regulatory cells like Tregs and myeloid-derived suppressor cells (MDSCs) [87]. These factors can inhibit the function of unconventional immune cells. For example, TGF-β can suppress the cytotoxic activity of γδ T cells, while Indoleamine 2,3-Dioxygenase 1 (IDO)-mediated tryptophan depletion can impair MAIT cell function [88,89]. Additionally, TGF-β can skew γδ T cells toward an IL-17-producing phenotype, which has been associated with tumor-promoting inflammation and angiogenesis rather than antitumor immunity [90,91]. Also, cancer-associated fibroblasts (CAFs) produce chemokine-driven immune exclusion by secreting C-X-C Motif Chemokine Ligand 12 (CXCL12), chemokine ligand 2 (CCL2), and Chemokine ligand 5 (CCL5), which can form physical and chemical gradients that prevent effective infiltration of cytotoxic lymphocytes [92,93].

### 5.4. Limited Persistence and Expansion In Vivo

Achieving sustained persistence and expansion of adoptively transferred unconventional immune cells, such as γδ T cells, MAIT cells, and invariant NKT cells, can be challenging [94,95]. Factors contributing to limited persistence include lack of homeostatic cytokine support, rapid differentiation into effector phenotypes, and susceptibility to apoptosis [96,97]. For instance, γδ T cells often exhibit poor in vivo expansion, necessitating exogenous cytokine support such as IL-15 or IL-7 [64]. Similarly, MAIT cells may require specific microbial-derived antigens for activation and proliferation, which are often absent in the TME [98]. Strategies like genetic modification to express cytokine receptors or co-stimulatory molecules are being explored to enhance their persistence [99].

**Figure 2:**
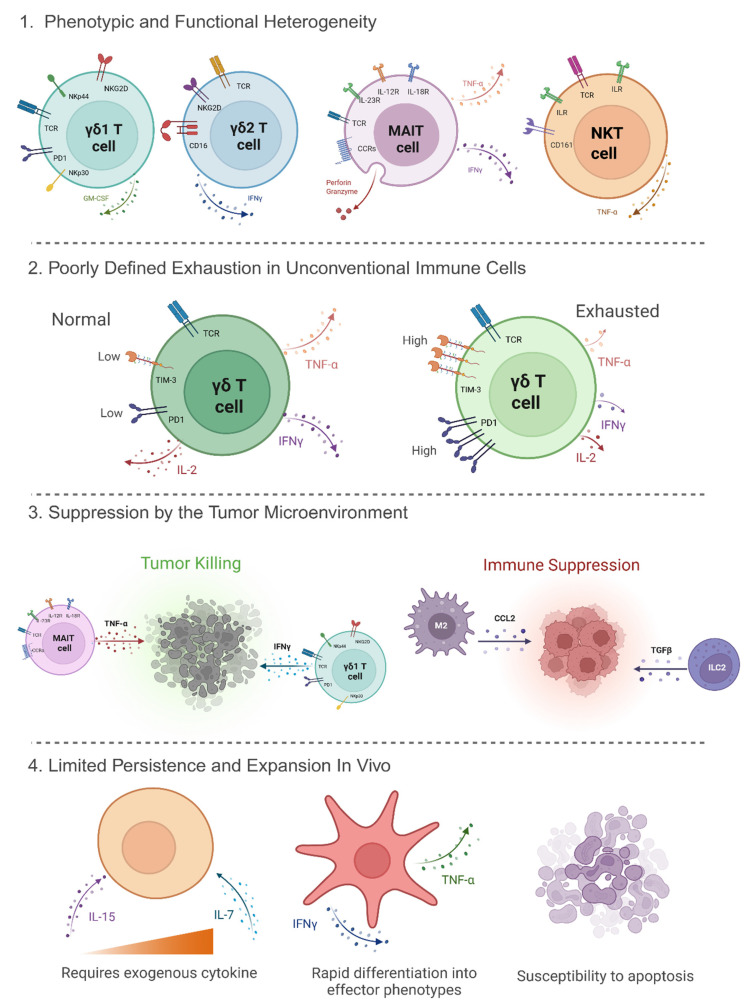
Major challenges limiting the efficacy of unconventional immune cells in cancer treatment. Created with BioRender.com.

Addressing these challenges requires a multifaceted approach, including detailed phenotypic and functional profiling of unconventional immune cells, elucidation of exhaustion pathways, modulation of the TME to reduce immunosuppression, and engineering strategies to enhance cell persistence and function [66,100]. Advancements in single-cell sequencing, gene editing, and synthetic biology hold promise for overcoming these translational hurdles and realizing the full potential of unconventional immunotherapies in cancer treatment [101]. The key challenges are summarized in Figure 2 and Table 1.

## 6. Off-the-Shelf Immunotherapies: Cross-Platform Potential

Off-the-shelf cancer immunotherapies signify a revolutionary change in cancer treatment, offering scalable and economical solutions by targeting shared antigens among patient groups. These methods eliminate the need for patient-specific customization by utilizing the MHC-independent cellular therapies and synthetic biologics to facilitate universal application [110]. In contrast to autologous approaches such as CAR-T cells, which are tailored to individual patients and require significant labor and time, allogeneic and synthetic forms provide scalable and accessible alternatives [110,111].

A significant benefit of allogeneic immunotherapies is their independence from MHC. Cell types, including γδ T cells, NK cells, and iNKT cells, function with minimal reliance on MHC presentation [110]. γδ T cells are a distinct subset of T lymphocytes, displaying a feature of both innate and adaptive immune cells, and they play a role in immunosurveillance of cancer. They offer an appealing alternative to traditional T-cell-based immunotherapy due to their lack of MHC restriction and their capacity to secrete elevated quantities of cytokines with established antitumor properties [103]. Moreover, they recognize stress ligands and phosphoantigens without classical antigen processing [103,112], allowing them to target a wide range of tumors with reduced risk of GvHD [113]. Similarly, NK cells provide the potential to serve as an allogenic therapy, as they neither necessitate stringent HLA compatibility nor entail the risk of GvHD. CAR-NK cells do not exhibit the same safety issues as CAR-T cells, including cytokine release syndrome noted in most CAR-T clinical trials [114,115]. iNKT cells bridge the innate and adaptive immune responses and exert antitumor effects through both cytotoxicity and immune modulation [116]. Despite the conceptual promise of iNKT cell-targeted immunotherapy, it encounters a significant technical obstacle for clinical implementation, mostly attributable to the low frequency of iNKT cells, especially in humans. To address this, it has been suggested to produce sufficient quantities of clinically proficient NKT cells from induced pluripotent stem cells for cancer immunotherapy [105].

Synthetic biologics, such as BiTEs and multi-specific antibodies, are a promising class of universal immunotherapeutics. These molecules redirect endogenous immune cells, typically CD3^+^ T cells, toward cancer cells by simultaneously binding tumor-associated antigens and immune effector receptors [117]. BiTEs have demonstrated clinical efficacy, particularly in hematologic malignancies, with notable examples like blinatumomab [118]. The modular design of multi-specific antibodies allows for the targeting of multiple tumor antigens or simultaneous engagement of co-stimulatory signals, enhancing tumor specificity and immune activation while minimizing off-tumor toxicity. Critically, these agents are cell-free and do not rely on patient-derived materials, which dramatically improves their shelf stability, production scalability, and global applicability [117,119].

Looking ahead, future strategies to enhance the safety, persistence, and adaptability of off-the-shelf immunotherapies include immune cloaking, advanced gene editing, and modular platforms. Immune cloaking techniques, such as HLA knockout using CRISPR/Cas9 (Clustered Regularly Interspaced Short Palindromic Repeats/ CRISPR associated protein 9), can render allogeneic cells less visible to host immune surveillance and reduce the risk of rejection, while overexpression of the “do not eat me” signal CD47 (integrin-associated protein) further protects these cells from phagocytosis by macrophages [120,121]. Advanced gene editing tools, including CRISPR/Cas9 and base editors, enable the precise removal of immunogenic components or insertion of beneficial transgenes to optimize cell function and reduce immunogenicity [108]. Additionally, modular platforms such as switchable CAR-T systems or universal immune effectors controlled by bispecific adaptors offer a plug-and-play approach that separates antigen recognition from effector function, enabling the same engineered cell product to be rapidly redirected to different targets through interchangeable components [122]. Collectively, these innovations promise to make off-the-shelf immunotherapies safer, more durable, and applicable to a broader range of patient populations.

This is a comparative table (Table 2) summarizing autologous versus allogeneic approaches, highlighting aspects such as manufacturing timelines, scalability, safety considerations, and emerging clinical outcomes.

## 7. Integrating Modalities and Personalization

The integration of various therapeutic modalities, patient stratification by multi-omics, and artificial intelligence (AI) are transforming cancer immunotherapy. Unconventional approaches, including neoantigen vaccines and engineered T cells, have shown synergistic potential when combined with immune checkpoint inhibitors, such as anti-PD-1 therapies [129,130]. Furthermore, Peng et al. [131] demonstrated in a mouse model of pancreatic cancer that neoantigen peptide vaccines induced tumor-specific T-cell responses and delayed tumor growth. However, the effect was augmented with dual T-cell immunoreceptor with Ig (immunoglobulin), T-cell immunoreceptor with Ig and ITIM domains (TIGIT), and PD-1 checkpoint blockade, resulting in increased functional CD4^+^ and CD8^+^ T-cell infiltration and improved tumor control. This result was further supported by translational studies using peripheral blood mononuclear cells (PBMCs) from vaccinated pancreatic ductal adenocarcinoma (PDAC) patients, where TIGIT blockade restored neoantigen-specific T-cell function, highlighting the potential of this combination immunotherapy strategy for clinical pancreatic cancer treatment. Another study demonstrated that genetically engineered γδ T cells secreting anti-PD-1 antibodies (Lv-PD1-γδ T cells) exhibit enhanced cytotoxicity and therapeutic efficacy against ovarian cancer compared to conventional γδ T cells. Their approach showed improved tumor suppression and increased survival rates and favorable safety profiles, highlighting Lv-PD1-γδ T cells as a promising strategy for cancer immunotherapy [132]. Clinical studies showed that combining T-VEC (Talimogene laherparepvec), an oncolytic virus, with pembrolizumab achieved higher response rates, up to 62%, in melanoma, compared to 33% with immune checkpoint inhibitors alone, accompanied by increased intra-tumoral CD8^+^ T cells and reduced immunosuppressive populations [133].

Patient stratification using multi-omics, such as integrating genomic, transcriptomic, epigenomic, spatial, and immunophenotypic data, facilitates optimal therapy selection by delineating tumor heterogeneity and immune landscape. For instance, high-throughput sequencing has revealed that patients with high tumor mutation burden (TMB) exhibit increased neoantigen production, enhancing immune cell recognition and response to immune checkpoint inhibitors [134]. Additionally, single-cell transcriptomics and proteomics have identified specific immune cell subsets, such as CD8^+^ T cells and cancer-associated fibroblasts, that influence immunotherapy efficacy [135,136]. Despite these advances, challenges remain in data integration and clinical validation, underscoring the need for standardized protocols and interdisciplinary collaboration.

To address translational challenges in unconventional immunotherapies, several actionable strategies are emerging. For MAIT cells, optimized ex vivo expansion using MR1 ligands (e.g., 5-OP-RU, a MAIT-activating ligand) and cytokines like IL-7 and IL-23, along with CRISPR-based CAR integration, enhances scalability and stability [15,26,102]. To improve the safety of cytokine fusions, tumor-conditional activation (e.g., protease-cleavable linkers) and antibody-tethered cytokines localize activity and reduce systemic toxicity [30,79,103,104]. γδ T-cell persistence can be increased via IL-15 co-expression, PD-1 knockout, or Vδ1 subset selection [137,138]. To counter the TME suppression, armored CARs expressing dominant-negative TGF-β receptors and chemokine receptor engineering (e.g., CXCR3 (Chemokine receptor CXCR3)) improve infiltration and function [29,64].

AI is also being concretely applied to multiple aspects of next-generation immunotherapy design. For instance, AI-driven platforms such as EVX-01, a personalized neoepitope vaccine, are being utilized to design personalized neoantigen-based cancer vaccines, which can be integrated with unconventional immunotherapies such as CAR-engineered γδ T cells or iNKT cells to enhance tumor-specific responses and overcome immune resistance in solid tumors [139,140]. Patient stratification based on multi-omics and spatial profiling is being conducted using integrative tools like PIONEER, which can guide the selection of optimal candidates for unconventional immunotherapies by identifying tumors enriched in stress ligands, non-classical antigen presentation (e.g., MR1, CD1d), or immunosuppressive microenvironments amenable to γδ T-cell-, MAIT-cell-, or iNKT-based interventions [141]. CAR design optimization for unconventional immune cells such as γδ T cells, MAIT cells, and iNKT cells can also leverage structural prediction tools like AlphaFold2 and ProteinMPNN, while deep learning-based tumor microenvironment modeling enables in silico prediction of synergistic drug combinations and immune cell performance, supporting the rational engineering of resilient and effective cell therapies in solid tumors [139,142].

AI is accelerating the translation of this complexity into practice by analyzing multi-modal data to predict immunotherapy responses and match optimal immune strategies to individual tumor landscapes. For instance, Cai et al. [141] demonstrated that AI-driven platforms can integrate multi-omics and histopathology to accurately predict immunotherapy response and guide bespoke vaccine or cell therapy design, outperforming traditional biomarkers such as PD-L1 status. Ongoing clinical trials are employing AI-powered platforms such as PIONEER and DeepChain to analyze patients’ tumor genomics and predict the most immunogenic neoantigens for personalized vaccine formulation, as reported in recent studies of EVX-01, in metastatic melanoma and TG4050, an individualized therapeutic vaccine, in head and neck cancer [139,143]. The EVX-01 Phase II study uses an AI-driven workflow to select vaccine targets, and preliminary results have shown enhanced T-cell responses and promising rates of tumor regression when combined with immune checkpoint inhibitors [143]. Similarly, the TG4050 trial utilizes NEC’s AI technology (a platform from NEC Corporation) to identify relevant neoepitopes based on individual HLA profiles, resulting in precise immunization with minimal off-target toxicity [144]. These advances underscore how integrating AI not only streamlines the personalization process but also holds the potential to improve both treatment outcomes and safety in cancer immunotherapy significantly. 

## 8. Novel Strategies to Overcome Translational Barriers

**Figure 3:**
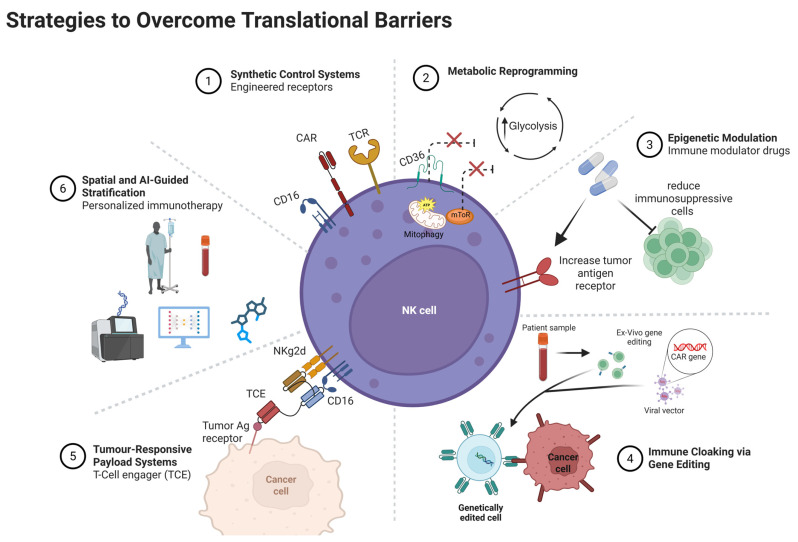
Innovative approaches and strategies have been developed to enhance the effectiveness of unconventional T-cell-based immunotherapies. Created with BioRender.com.

The limited use of immunotherapies has been addressed, which has led to the emergence of different novel approaches that would overcome such translational challenges, which are summarized in Figure 3.

### 8.1. Synthetic Control Systems

The concept of cancer immunotherapy relies on inducing robust antitumor immune responses that can eradicate the tumor and prevent its recurrence via generating long-lived memory T cells [145]. Various cancer immunotherapy approaches have been developed and used in clinical trials or even for treating cancer patients. These approaches include immune checkpoint inhibitors, peptide-based vaccines, protein-based cancer immunotherapy, oncolytic viruses, and adoptive T-cell transfer (ACT) [145,146,147,148,149]. CD8^+^ T cells represented in the ACT strategy are the milestone of cancer immunotherapy due to their ability to specifically recognize and kill tumor cells by the ligation of TCR with the tumor antigen peptide presented on the MHC. Endogenous CD8^+^ T-cell response most often demonstrates impaired function, and it is insufficient to protect against an established tumor due to the immunosuppressive tumor microenvironment [150,151]. Thus, researchers have transitioned towards novel strategies to engineer TCRs to overcome this obstacle. This can be achieved by increasing the binding affinity between the engineered TCR and tumor antigens [145]. Currently, there are two common approaches for T-cell immunotherapy: CAR T cells and TCR-engineered T cells. The major challenge for this approach is to find an appropriate tumor-associated antigen (TAA) that is exclusively expressed on tumor cells to avoid autoimmune diseases, with strong binding affinity to αβ-TCR to initiate robust TAA-specific CD8^+^ T-cells responses and long-lived memory T cells [152]. CAR-T cells are generated in the laboratory following the collection of patients’ blood. T cells are then isolated, and the gene for the specific antigen is inserted into T cells using viral or non-viral vector systems. The engineered cells are expanded and then fused back into the patient’s bloodstream [153]. CAR-T cells are designed to express single-chain variable fragments (scFv) of a mAb (antigen binding domain) directed to a specific TAA and linked with a co-stimulatory receptor such as CD28 (co-stimulatory molecule), 4-1BB (CD137, an inducible costimulatory receptor), OX40 (CD134, Tumor necrosis factor receptor superfamily, member), CD40 (type I transmembrane protein), inducible T-cell co-stimulator (ICOS), CD27 (member of the tumor necrosis factor receptor superfamily) and CD3zeta chain, or T-cell surface glycoprotein CD3 zeta chain for full CAR T-cell activation [153,154,155]. CARs are HLA-independent, meaning that they recognize native proteins found on the cell surface, thus overcoming limitations imposed by HLA-restriction of TCRs [151].

CAR T cells showed promising results in the clinic against multiple blood cancers like leukemia, lymphoma, and multiple myeloma but failed in treating solid tumor [6]. On the other hand, the TCR engineering approach relies on identifying TCRs that recognize HLA-presented peptide fragments derived from proteins of all cellular compartments [151]. Similar to the CAR-T-cell approach, TCRs with specific binding to TAA are isolated, sequenced, expanded ex vivo, and injected back into the same patients to fight cancer. This approach showed promising clinical results in multiple cancer models, including melanoma, sarcoma, and neuroblastoma [156,157,158,159]. Although CAR T cells and TCR engineering are showing promising results in cancer immunotherapy, there are some barriers that can limit their full potential, including TCR mismatch of exogenous TCR αβ gene sequence with the endogenous genes introduced by genetically engineered T cells. This might result in generating a T-cell pool that may recognize and attack self-antigens, leading to autoimmune diseases [160]. Antigen escape is a common tumor resistance mechanism, providing a real challenge for CAR-T-cell therapy. In addition, the harsh conditions of the tumor microenvironment make it difficult for TAA-specific CD8^+^ T cells to infiltrate the tumor, and they can be suppressed directly by the ligation of co-inhibitory receptors expressed on activated CD8^+^ T cells with their ligands on tumor cells or indirectly by suppressor cells such as T regulatory cells and myeloid-derived suppressor cells. Cytokines like IL-10 and TGF-β also play a major role in suppressing effector CD8^+^ T cells. Cytokine storm resulting from overactivation of transferred CAR-T cells has also been reported in some patients, and it should be highly considered and managed carefully to avoid undesirable, lethal adverse events [155]. Thus, numerous studies are being conducted to overcome these challenges to improve the efficacy of ACT treatment.

### 8.2. Metabolic Reprogramming

Accumulated evidence collected throughout the past years has emphasized the crucial impact of cellular metabolism on T-cell development, proliferation, and differentiation into effector and memory T cells. Each phase relies on a specific metabolic pathway to meet its needs [161]. For example, during naïve and memory T cells, they use energy-efficient oxidative metabolism, while proliferating T cells shift to highly glycolytic metabolism to meet their bioenergetic demand during the proliferation process [161]. In the tumor microenvironment, the amount of available metabolites, which normally remains stable and at an adequate level in healthy tissues, is unbalanced and is under frequent fluctuation [162].

Tumor infiltrating lymphocytes (TILs) are in a constant competition with tumor cells for key nutrients, glucose, and oxygen. These harsh conditions result in CD8^+^ T cells’ hyporesponsiveness regardless of the immunogenicity of the tumor antigen or even when strong stimuli are applied to boost their effector function [163]. Thus, targeting the metabolic pathway in TILs has been considered a promising approach to restore their vitality for more effective cancer immunotherapy [164]. For example, enhancing fatty acid oxidation (FAO) of TILs in melanoma partially preserved CD8^+^ T cells’ effector functions and slowed tumor progression [165]. Moreover, it has been reported that tumor-infiltrating CD8^+^ T cells rely on lipid as an alternative metabolic pathway to compensate for the limited amount of available glucose within the tumor microenvironment to remain functioning. However, excessive uptake of lipids can lead to lipotoxicity and induce ferroptosis in TILs. CD36 (type 2 cell surface scavenger receptor), which is responsible for lipid transportation, was found to be highly expressed on tumor infiltrating CD8^+^ T cells, promoting intratumoral CD8^+^ T-cell dysfunction [166]. Thus, damping CD36 function either by knocking out the gene or by using anti-CD36 restored CD8^+^ T cells’ effector function can solve this issue [167]. In 2021, S Xu et al. investigated the effect of knocking down the CD36 gene in a tumor-bearing mice. The study revealed that CD36^−/−^ CD8^+^ T cells enhanced tumor regression and restored cytotoxic T lymphocytes (CTL) effector function and cytokine production [167].

Active mammalian target of rapamycin (mTOR) is a protein kinase that plays a major role in promoting glycolysis. This protein kinase serves as an important factor for regulating multiple biological processes, including cellular metabolism, regulating cell growth, autophagy, survival, proliferation, migration, and protein synthesis [168,169,170]. In addition to the mTOR role in glycolysis, it has been reported that Phosphoinositide 3-kinases (PI3K)/Protein kinase B (Akt)/mTOR contributes to shaping the interaction between TILs and tumor cells, suggesting the key role of mTOR in determining tumor growth, progression, and drug resistance. Regulating the mTOR signaling pathway (by inhibiting glycolysis) showed strong antitumor immune responses through promoting the development of memory CD8+ T cells that can replicate rapidly upon second exposure to tumor antigen, allowing more sufficient clearance of tumor cells [171,172]. Thus, targeting mTOR could be an encouraging approach for developing anticancer drugs. Other strategies have been tested to mitigate T-cell senescence, including activating mitochondrial mitophagy, regulating the balance of antioxidants, and regulating mitochondrial dynamics [173]. Finally, altering CD8^+^ TILs’ metabolism in combination with immune checkpoint inhibitors may result in better therapeutic outcomes [174], suggesting that combination therapy might be more beneficial in treating cancer compared to monotherapy.

### 8.3. Epigenetic Modulation

Epigenetic modulators such as deoxyribonucleic acid (DNA) methyltransferase inhibitors and histone deacetylase inhibitors have been used for decades as monotherapies or in combination with chemotherapy for cancer treatment. However, their combination with immunotherapies has not yet been approved by regulatory agencies [175,176]. Combining epigenetic modulators with immunotherapy could synergistically enhance therapeutic effects through several mechanisms. These include enhancing tumor immunogenicity by making tumors more visible to the immune system. For instance, the Phase Ib NIBIT-M4 study, which evaluated guadecitabine (a DNA methyltransferase inhibitor) combined with ipilimumab (a CTLA-4 inhibitor), demonstrated increased expression of tumor antigens and HLA molecules in melanoma patients [177]. Additionally, epigenetic drugs can reduce immunosuppressive cells such as myeloid-derived suppressor cells and regulatory T cells while promoting the infiltration of immune effector cells. In the Phase I ETCTN-9844 trial, the histone deacetylases (HDAC) inhibitor entinostat combined with nivolumab (a PD-1 inhibitor) ± ipilimumab was tested in 33 patients with advanced solid tumors. This regimen increased the intratumoral CD8^+^/Treg ratio and significantly reduced the frequency of PD-L1-expressing MDSCs [177]. While combining epigenetic therapies with immunotherapy holds therapeutic potential for numerous cancers, it may also increase the risk and severity of immune-related adverse events, such as colitis, diarrhea, and hepatitis [177,178]. Furthermore, multi-omics profiling is indispensable for identifying predictive molecular signatures to optimize patient selection, enhancing both the safety and efficacy of epigenetic–immunotherapy combinations and facilitating future clinical translation [179].

Beyond cellular and pharmacological approaches, lifestyle and environmental factors are increasingly recognized for their role in modulating epigenetic processes, which subsequently influence immune function and antitumor responses. Nutrition, meditation, stress reduction, and sunlight or red-light exposure have been shown to induce favorable epigenetic changes and enhance antitumor immunity [180,181]. Nutrients like ginger and high-dose ascorbic acid (vitamin C) also exhibit epigenetic and direct anticancer effects [182,183]. Additionally, dimethyl sulfoxide (DMSO), a well-known solvent with anti-inflammatory and antioxidant properties, has demonstrated promising antitumor potential and may serve as a safe adjuvant in future clinical studies [184]. These accessible, low-risk strategies synergize effectively with metabolic reprogramming approaches like fasting and glucose restriction, positioning them as valuable components of integrated immunotherapeutic frameworks.

### 8.4. Immune Cloaking via Gene Editing

Immunotherapy harnesses the immune system to target cancer cells, achieving breakthroughs, particularly in blood cancers, through CAR T-cell therapy and checkpoint inhibitors. However, it faces challenges in treating solid tumors due to immune evasion mechanisms. These include antigen downregulation, the creation of a physically and chemically immunosuppressive microenvironment, and active suppression of infiltrating immune cells. Additionally, risks such as GvHD from donor-derived cell therapies can limit their persistence and efficacy [185]. Immune cloaking via precise gene editing has gained significant traction in recent years as a strategy to overcome immunotherapy resistance by eliminating or modifying key immunogenic surface proteins on therapeutic cells. This approach confers protection against both tumor-induced immunosuppression and host-mediated clearance [186]. In the Phase I clinical trial of ALLO-316, an allogeneic CAR T-cell therapy, the Transcription Activator-Like Effector Nuclease (TALENs) gene-editing technique was used to knock out the TCRα and the CD52 (a small glycoprotein that suppresses T-cell activation) genes in patients with clear-cell renal cell carcinoma (ccRCC). Preliminary results have been promising, with no reported cases of GvHD to date; the primary completion date is expected by the end of 2025 [187]. However, several challenges remain for the broader application of immune cloaking via gene editing. Gene-edited cells may lose their cloaking capabilities over time due to gene reactivation or host immune adaptation. Furthermore, the scalable production of multi-gene-edited cells is hindered by high costs and complex manufacturing processes [108,188].

### 8.5. Tumor-Responsive Payload Systems

Tumor-responsive payload systems are an advanced strategy to improve immunotherapy specificity and safety by restricting activation to the tumor microenvironment. By exploiting tumor microenvironment-specific cues, such as proteases or acidic pH, prodrugs and engineered oncolytic viruses can localize immune activation and reduce systemic toxicity while enhancing efficacy. These approaches have shown promise in preclinical and early clinical studies. A key example of a prodrug-based approach is the protease-activated T-cell engager (TCE), which is masked by a protease-cleavable peptide to prevent CD3 binding in healthy tissues. In the tumor microenvironment, elevated protease activity cleaves the masking peptide, enabling TCE activation. JANX007 exemplifies this strategy by targeting prostate-specific membrane antigen (PSMA) on tumor cells and CD3 on T cells [189]. In the case of oncolytic viruses, the ongoing BT-001 Phase I/IIa trial (NCT04725331) utilizes oncolytic viruses engineered to locally express an anti-CTLA-4 antibody and GM-CSF within solid tumors. The virus replicates selectively in tumor cells, resulting in intratumoral payload expression, enhanced immune cell infiltration, and modulation of the tumor microenvironment [190].

### 8.6. Spatial and AI-Guided Stratification

The use of AI is being increasingly integrated into the field of immunotherapy, providing unprecedented opportunities to predict patients’ response to a given anticancer drug, enhance the treatment efficacy, and discover novel tumor targets [191,192]. Results for using cancer immunotherapy products are noticeably variable among patients, where some patients experienced a 100% response rate with complete tumor regression, while others may have partial or no responses. This is largely due to the complex and heterogeneous nature of tumors [191,192]. Using AI privilege in analyzing large and complex biological data, it is now possible to overcome the limitations associated with the traditional use of immunotherapies and to refine these therapies, resulting in an enhanced effect, predicted patient response, and accelerating novel immunotherapeutic discovery. For example, tissue biomarkers analyzed by AI-based digital pathology discover new information that may not be predicted by humans, and using this information can yield more accurate tumor diagnosis, formulate treatment plans, as well as provide an insight into patients’ progress to a specific treatment, reducing the cost and workload associated with conventional trial-and-error approaches [142,193,194]. AI has revolutionized personalized treatment by identifying novel biomarkers that can predict the effectiveness of immunotherapy more rapidly than the current approaches, by rapid analysis of large-scale genomics and proteomics data, which is critical for enhancing the outcomes [194]. However, to avoid misleading information, the data provided for this approach must be comprehensive and precise [144].

## 9. Conclusions

Unconventional immunotherapies are emerging as disruptive innovations within the cancer treatment landscape, particularly in addressing the limitations of conventional strategies in solid tumors, where therapeutic efficacy has historically been limited. Utilizing nonconventional immune cell populations and engineered biologics, such biotherapeutic platforms, have unique immunologic advantages such as MHC-independence, tissue residency, rapid cytotoxicity, and bypassing tumor immune-evasion and immune-resistance mechanisms. Approaches including allogeneic γδ T cells, iNKT cells, and modular multi-specific antibodies are currently increasing in availability, providing a paradigm-shifting opportunity to democratize immunotherapy to global populations. But their success relies on sustained efforts in elucidating mechanistic fundamentals, optimizing synthetic engineering, and establishing reliable manufacturing pipelines. Combining with AI-augmented patient stratification, multi-omics, and tumor microenvironment mapping will make these therapies even more personalized. Finally, non-conventional immunotherapies are not only alternatives but rather new agents with the potential to surpass the standards with an aim to enhance the curative responses in cancers that were previously considered untreatable.

## Figures and Tables

**Table 1:** Translational Challenges, Engineering Strategies, and Clinical Status of Unconventional Cell-Based Immunotherapies.

Modality	Translational Challenges	Engineering Strategies	Clinical Status	References
CAR-MAIT Cells	Limited ex vivo expansion; low frequency in peripheral blood; MR1 restriction	Feeder-free expansion using 5-OP-RU and IL-7/IL-23; CRISPR-mediated CAR knock-in with inducible promoters	Preclinical studies	[15,26,102]
CAR-γδ T Cells	Short in vivo persistence; donor variability; functional exhaustion	IL-15 co-expression; PD-1 knockout; Vδ1 subset selection; CXCR3 chemokine receptor engineering; metabolic support	Phase I trials are ongoing, ADI-270 (renal cell carcinoma) and KB-GDT-01 (metastatic non-small-cell lung cancer) received *FDA Fast Track* status	[30,79,103,104]
CAR-iNKT Cells	Low abundance in humans; need for sustained effector function	Use of IL-15-expressing CARs; CD62L^+^ donor selection; IL-12 polarization; scalable iPSC-derived iNKT generation	Phase I trial completed in neuroblastoma (25% ORR, no DLTs); not yet FDA-approved	[19,21,22,105]
CAR-Tregs	In vivo instability; risk of off-target suppression; antigen specificity	FoxP3-stabilized CAR constructs; antigen-specific Treg targeting; local delivery using synthetic biology control systems	Preclinical studies are promising for autoimmunity and transplantation	[17,32,106]
Double-Negative T Cells	Low abundance; insufficient expansion protocols; unclear clinical niche	Leverage TNFα-JAK1-ICAM-1 cytotoxic axis; GvHD-free cytotoxic approaches for AML	Preclinical only: functional studies in AML mouse models	[24,31,107]
Universal CAR Platforms	Immunogenicity of allogeneic cells: risk of rejection and poor persistence	CRISPR-mediated HLA-I/II knockout; CD47 overexpression (“do not eat me” signal); modular switchable CAR constructs for universal applicability	Early-phase clinical trials: hypoimmunogenic CAR-Ts show early safety data	[18,108,109]

**Table 2:** Summary of autologous versus allogeneic approaches, highlighting aspects such as manufacturing timelines, scalability, safety considerations, and emerging clinical outcomes.

Feature	Autologous Immune Cell Therapies	Allogeneic “Off-the-Shelf” Immune Cell Therapies	References
Source of Cells	Patient-derived (e.g., T cells, NK cells)	Healthy donors or universal engineered cell lines	[123,124,125]
Manufacturing Time	Several weeks due to patient-specific cell collection and expansion	Pre-manufactured and cryopreserved; ready for immediate use	[123,126,127]
Scalability	Limited by patient-to-patient production	Highly scalable; batch production for multiple patients	[124,125,126]
Cost	High due to individualized manufacturing	Potentially lower due to economies of scale	[123,125,126,127]
Consistency and Quality Control	Variable, dependent on patient health and immune status	More uniform product with standardized quality	[123,125,127]
Risk of Immune Rejection	Minimal, as cells are self-derived	Higher risk; requires gene-editing to remove alloreactive components (e.g., TCR knockout, HLA engineering)	[123,124,126,128]
Graft-versus-Host Disease (GvHD) Risk	Low (self-cells)	Higher risk unless modified (e.g., using gene editing or NK cells/γδ T cells)	[123,124,125,126]
Time to Treatment	Delayed (weeks for manufacturing)	Immediate (on-demand use)	[123,127]
Applications in Current Trials	CAR-T (CD19, BCMA), TILs	Universal CAR-T (UCART19, ALLO-501), CAR-NK, engineered γδ T cells	[123,126,127,128]
Integration with Gene Editing	Limited (focus on persistence, safety switches)	Essential (to overcome host rejection, enhance tumor targeting, and remove alloreactive responses)	[126,128]

## Data Availability

No new data were created or analyzed in this study.
